# A Simple and Non-Invasive Method for Nuclear Transformation of Intact-walled *Chlamydomonas reinhardtii*

**DOI:** 10.1371/journal.pone.0101018

**Published:** 2014-07-02

**Authors:** Sora Kim, Young-Chul Lee, Dae-Hyun Cho, Hyun Uk Lee, Yun Suk Huh, Geun-Joong Kim, Hee-Sik Kim

**Affiliations:** 1 University of Science and Technology (UST), Daejeon, Republic of Korea; 2 Department of BioNano Technology, Gachon University, Seongnam-si, Republic of Korea; 3 Environmental Biotechnology Research Center, Korea Research Institute of Bioscience & Biotechnology, Daejeon, Republic of Korea; 4 Division of Materials Science, Korea Basic Science Institute, Daejeon, Republic of Korea; 5 Department of Biological Engineering, College of Engineering, Inha University, Incheon, Republic of Korea; 6 Department of Biological Sciences, College of Natural Sciences, Chonnam National University, Gwang-ju, Republic of Korea; Northwestern University Feinberg School of Medicine, United States of America

## Abstract

Genetic engineering in microalgae is gaining attraction but nuclear transformation methods available so far are either inefficient or require special equipment. In this study, we employ positively charged nanoparticles, 3-aminopropyl-functionalized magnesium phyllosilicate (aminoclay, approximate unit cell composition of [H_2_N(CH_2_)_3_]_8_Si_8_Mg_6_O_12_(OH)_4_), for nuclear transformation into eukaryotic microalgae. TEM and EDX analysis of the process of transformation reveals that aminoclay coats negatively-charged DNA biomolecules and forms a self-assembled hybrid nanostructure. Subsequently, when this nanostructure is mixed with microalgal cells and plated onto selective agar plates with high friction force, cell wall is disrupted facilitating delivery of plasmid DNA into the cell and ultimately to the nucleus. This method is not only simple, inexpensive, and non-toxic to cells but also provides efficient transformation (5.03×10^2^ transformants/µg DNA), second only to electroporation which needs advanced instrumentation. We present optimized parameters for efficient transformation including pre-treatment, friction force, concentration of foreign DNA/aminoclay, and plasticity of agar plates. It is also confirmed the successful integration and stable expression of foreign gene in *Chlamydomonas reinhardtii* through molecular methods.

## Introduction

Genetic engineering in prokaryotic unicellular organisms has reached newer heights with metabolic and pathway engineering, sometimes leading to drastic change in the identity of the parent strain [1], [2]. However, eukaryotic unicellular organisms like microalgae pose several bottlenecks to genetic engineering, foremost among them is transformation [3]–[5]. The presence of nuclear genome followed by organellar genomes like chloroplast and mitochondria has not helped for smooth entry of foreign DNA into targeted genome. Hence there has been a need for specific transformation methods for each genome and nucleus transformation has been the most difficult to achieve because of the resistance of two membranes, cell wall/membrane followed by nuclear membrane [6], [7]. In essence, the rupture of the tough algal cell wall and the aforesaid membrane followed by successful integration and above all, survival of the cell to generate transformed progeny is a delicate exercise, which points to the handful of protocols with low efficiencies and high sophistication [8], [9].

Among the various methods established, agitation with glass beads needs protoplast generation [3]–[5], silicon carbon whiskers based-method has been reported to be toxic to human [5], [6], electroporation [5]–[7], [9] and biolistic microparticle bombardment [5]–[7] needs expensive instrumentation and protoplast generation, and *Agrobacterium tumefaciens*-mediated gene transfer is inefficient [5]–[8], [10]. Hence, in spite of such efforts, genetic engineering in eukaryotic microalgae is not well-established, compared with bacterial transformation [2], [5], [7], [11]–[13]. The development of simpler and more efficient methodology is needed.

Since its synthesis [14], aminoclay has been used to construct (nano) composites of biomolecules such as DNA [15], [16], proteins [17], [18], and phospholipid [19]. Organic-inorganic nanocomposites of aminoclay have also been employed as antimicrobial agents [20], [21], as additives in bacterial transformation [13], as a selective lytic-agent of red tide-causing microorganisms [22], and as an efficient microalgae-harvesting agent [23], [24]. For the extended synthesis of aminoclay family, magnesium (Mg) based aminoclay showed smaller average particle size (∼45 nm) with a narrow distribution [22] rather than calcium (Ca) [25], iron (Fe) [26], and copper (Cu)-aminoclay [27] with all approximate hydrodynamic particle size at>100 nm, indicating Mg-aminoclay possess transparent and water-soluble properties [28] and acts as effective interactions of organism’s cells [13], [22]–[24], [29]. Thus, in size viewpoint of aminoclay, Mg-aminoclay also can be a strong candidate for nuclear transformation of microalgal cells in the present study. For the brevity of terms, herein Mg-aminoclay indicates ‘aminoclay’.

Because of the attractive characteristics of aminoclay, such as its low toxicity profile with regards to environment [30] and mammalian cells [25], we have used it to develop a method for transformation in intact *Chlamydomonas reinhardtii* cells. This method provides a simple, powerful, and non-invasive tool to transform cell wall-intact algal cells, without delicate pre-treatment such as enzymatic digestion and overcoming efficiency bottleneck that has limited genetic engineering of algal cells through nuclear transformation.

## Materials and Methods

The detailed materials and equipments of aminoclay synthesis, *C. reinhardtii* culture, *C. reinhardtii* transformation, DNA extraction, PCR analysis, TEM examination, spreading friction apparatus, and commercially available kits used in this study are displayed in Method S1 in File S1.

### Reagent setup

The detailed Tris-Acetate-Phosphate (TAP) salts solution, liquid and solid TAP medium, and medium A (4% agar with Hygromycin B) and medium B (1.5% agar with Hygromycin B) are described in Text S1 in File S1.

### Spreading friction equipment setup

Secure the experimental stand, ∼1 meter height, on the desk with a clamp. Under the clamp, place a rotor with control buttons (speed and timer) and ensure it is level. Hang a triangle shaped polystyrene stir stick to a force control gauge that is perpendicularly tightened to the experimental stand (Figure S1 in File S1).

### Preparation of aminoclay

According to literature [22], [28], [31], use a magnetic stirrer to dissolve 8.4 g of MgCl_2_•6H_2_O into 200 ml bulk ethanol solution in 500-ml beaker for 20 min. Add 13 ml 3-aminopropyl triethoxysilane to this bulk ethanolic solution and stir for 12 hours. Collect white-colored products by centrifugation at 6000 *g* for 10 min. Rinse the precipitate products two times with 200 ml bulk ethanol. Dry the harvested aminoclay on the oven at 50°C for 24 hours in order to let evaporation of the residual ethanol solvent. Grind dried aminoclay lumps using a mortar and pestle.

### Cultivation and harvesting of *C. reinhardtii* CC-124 wild type mt- [137c] (nit1 mutation)

Based on the literature [32], inoculate cells at a density of 1×10^5^ cells ml^−1^ in 1 liter of TAP liquid medium and grow at 25°C, under constant agitation at 10 *g* force continuous white light (100 µE m^−2^s^−1^) for 3–5 days. Harvest the cells (1×10^8^ cells ml^−1^) by centrifugation (13,000 *g*; 1 min, 25°C).

### Optimization of *C. reinhardtii* transformation

Re-suspend 500 µl cell pellet to 500 µl mixture of aminoclay-DNA solution in a 1.5 ml-Eppendorf tube. Pre-treat the samples by vortexing for 10, 30, 60, 120, and 180 s (**Option A**) or tip ultrasonication for 5, 10, 20, and 30 s (**Option B**). Test mechanical properties (springiness, hardness, gumminess) of 1.5, 3.0, and 4.0% agar plates. Pipet 100, 200, or 300 µl aliquots of cell and DNA-aminoclay mixture solution, generated by vortexing (**Option A)** or tip ultrasonication (**Option B)** on 4.0% agar plates containing hygromycin B (15 µg/ml). Rub them using spreading friction equipment for 4 min at a constant speed of 0.22 *g* force.

### Measurement of zeta potential and hydrodynamic size with TEM characterization

Zeta potentials and hydrodynamic sizes of aminoclay (100 mg/mL), DNA (150 ng/µL), and DNA and aminoclay mixture (150 ng/µL in 100 mg/mL of aminoclay solution) were measured by dynamic light scattering (DLS) methods (Zetasizer nano zs, Malvern, UK) in triplicate.

To examine DNA and aminoclay hybrids *in vitro*, mix 500 µl hydrated aminoclay with DNA solution (at least 30 ng) in a 1.5 ml-Eppendorf tube. Drop 10 µl aliquot of the mixture of DNA-aminoclay solution onto a carbon mesh film copper grid. Dry the sample on the copper grid overnight at room temperature. Inspect it by TEM. To inspect the ultrastructural studyfor cross-sectioned cells [33], fix cells before and after vortexing, ultrasonication, or spreading, in 2.5% paraformaldehyde-glutaraldehyde mixture buffered with 0.1 M PBS solution (pH 7.2) for 2 hours. Post-fix in 1% osmium tetroxide in the same buffer for 1 hour and dehydrate in graded ethanol and propylene oxide. Embed in Epon-812 resin. Make ultra-thin sections by ultracut microtome, stain with uranyl acetate and lead citrate, and inspect under TEM.

### Cultivation of transformation-treated cells in the selected medium and picking the transformant colonies

Incubate cells at room temperature under static conditions and continuous light (60 µE m^−2^s^−1^) for 5–7 d. Pick the colonies and plate each one into a well of a 96-well plate containing medium A supplemented with Hygromycin B (10 µg µl^−1^). Transfer the transformant colonies and plate each one into a well of a 24-well plate containing medium B supplemented with Hygromycin B (20 µg µl^−1^). Every data point should be based on at least three independent experiments, and expressed as an average of the individual values with standard deviation.

### PCR experiment and Southern blot assay

Denature at 94°C for 5 min, followed by 35 cycles of 94°C for 45 sec, 59°C for 45 sec, 72°C for 1 min, ending with a final extension at 72°C for 7 min. PCR was performed in a thermal cycler (GeneAmp PCR System 2700, Applied Biosystems, USA, www.appliedbiosystems.com). Using a DNA extraction kit (i-genomic DNA Extraction Mini Kit for Plant-iNtRON), it is confirmed the colony identity by checking the insertion of plasmid DNA by PCR. It is noted that when considering the high GC contents of *Chlamydomonas* genome, PCR efficiency can be improved by adding DMSO and 5 M of Betaine or by using high GC buffer to the PCR mix.

Southern blotting assay was followed [34]. Briefly, after digestion of genomic DNA with *Kpn*I, the digested DNA was electrophoresed on 1% agarose gel and transferred onto a Hybond N^+^ membrane. PCR fragment of the C-terminal region of *aph7”* was probed. ^32^P-labeled probes were generated by the Rediprime Π Random labeling system (Amersham Biosciences, Piscataway, NJ). According to manufacturer’s guideline, the hybridization was conducted. Resultant signals were detected by the Bio-Imaging Analyzer BAS-1800 Π (Fuji, Tokyo, Japan).

## Results

### Aminoclay-based spreading friction method

**Figure 1:**
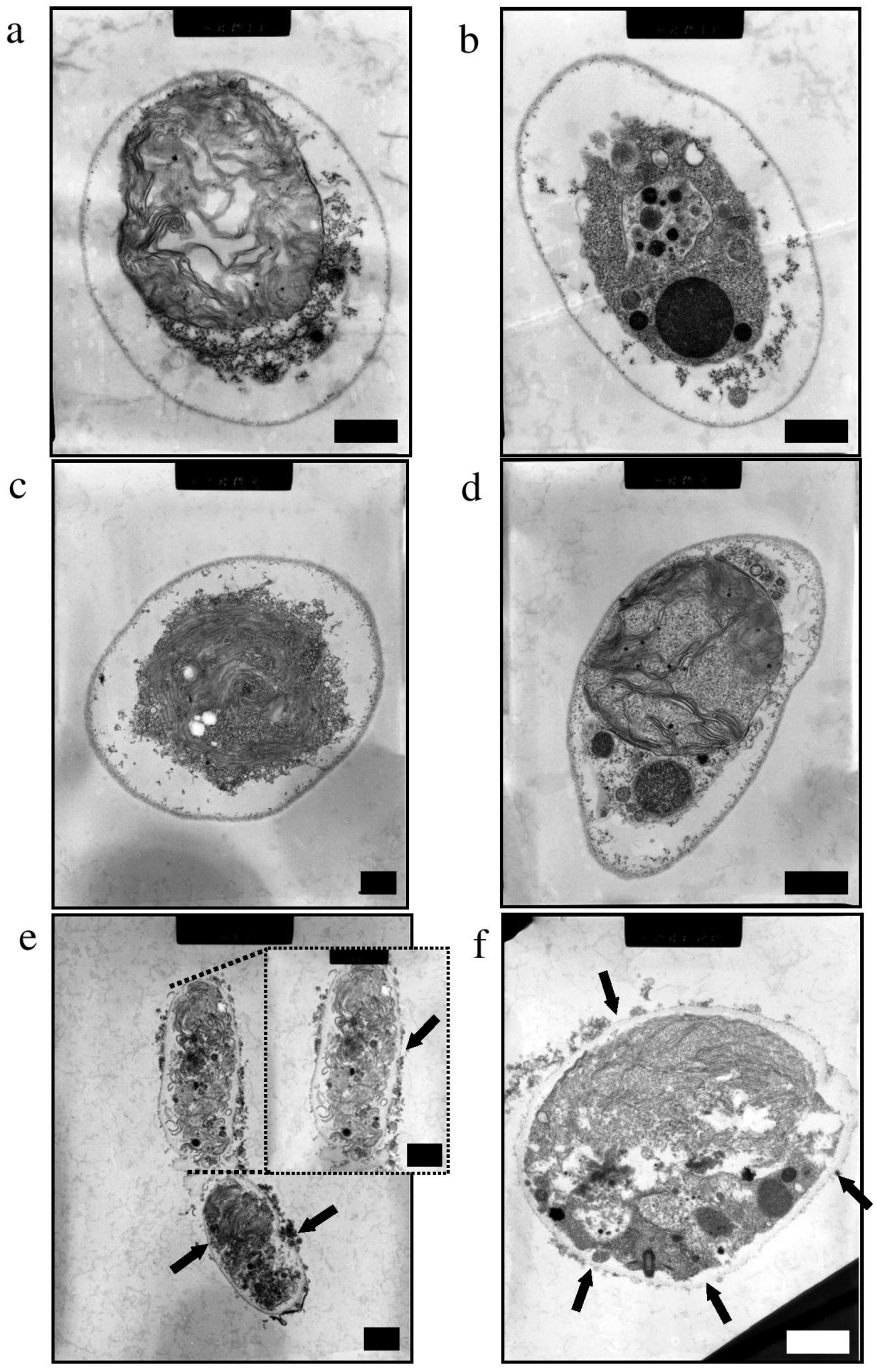
Cross-sectioned transmission electron microscopy (TEM) images. (a, b) intact cells, (c) cells after pre-treated vortexing or (d) ultrasonication with aminoclay-DNA hybrid for 60 s, and (e, f) cells pre-treated with aminoclay-DNA hybrid after spreading with a stir bar on an agar plate (4.0%). The inset in the image (e) shows a cell at high magnification. Black arrows (in e and f) indicate damaged or fractured cell walls. All scale bars = 1 µm.

Microalgal transformation is difficult to achieve because of the complex cell wall structures. Although DNA adsorbed nanofibers have been developed for bacterial transformation, popularly referred to as Yoshida effect, they proved insufficient for microalgal transformation in our earlier experiments (Figure S2a in File S1). Our earlier experiments also demonstrated that established methods for microalgal transformation like glass beads are ineffective without pretreatment (protoplast generation) (Figure S2b in File S1). Hence, the real challenge for transformation in microalgae was not only usage of the better materials but also selective rupture of cell membrane while keeping the cell functioning to generate progenies. We also hypothesized that an approach based on aminoclay-wrapping of DNA molecule could be more efficient than DNA adsorption onto inorganic fibers for transformation of algal cells because self-assembled DNA biomolecules by aminoclay wrapping can reach into the cytoplasm of cells more safely. Therefore, organo-building blocks of aminoclay (Figure S3 in File S1) were used to coat DNA molecules and produce an ultra-thin hybrid structure [15]. To achieve the effective coating of aminoclay on the DNA molecules, it was studied differing concentrations of aminoclay and different protocols for complete mixing and coating. Two techniques were employed for pretreatments viz., ultrasonication and vortexing (Table S1 in File S1). However, initial experiments with either ultrasonication or vortexing didn’t result in transformants. TEM analysis of the ultrastructural study revealed that cell damage was not sufficient enough for DNA delivery (**Figure 1a–d**). Further experiments were conducted to facilitate rupture of the cells. When sufficient friction force was applied to the sample mixture (containing aminoclay coated DNA) while spreading on the agar plate, transformants appeared. Although, the efficiency of transformation was low, TEM analyses confirmed the rupture of cell wall, as shown by pits (marked by arrows), and DNA delivery inside the cell (**Figure 1e–f**). All the parameters in the protocol were then optimized to achieve high efficiency.

### *In- vitro* characteristics of aminoclay, DNA, and aminoclay-DNA hybrid

**Figure 2:**
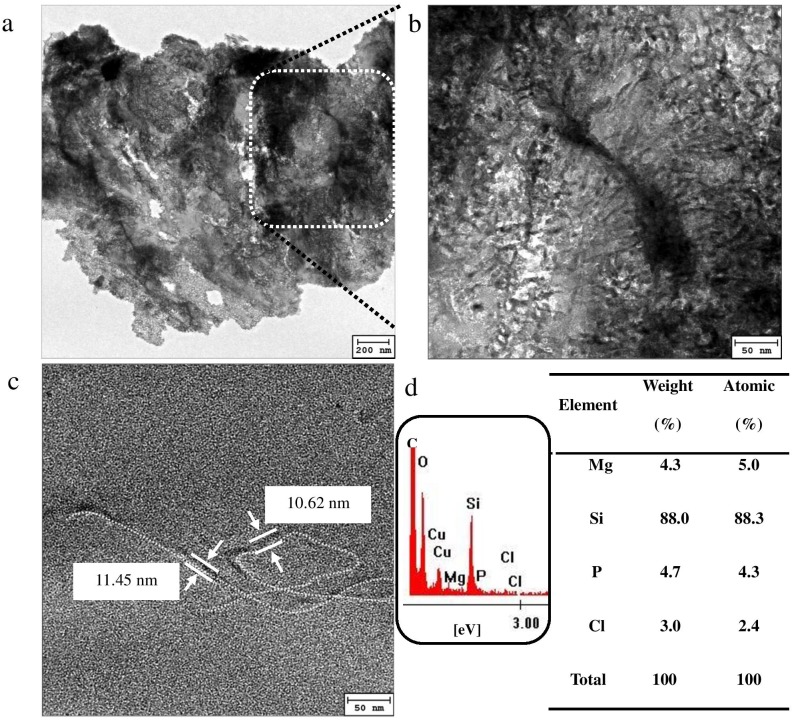
Transmission electron microscopy (TEM) images. (a) low and (b, c) high magnifications of aminoclay-DNA hybrid, and (d) energy-dispersive X-ray (EDX) spectrum of aminoclay-DNA hybrid with percentage atomic weights of magnesium (5.0%), silicon (88.3%) and chloride (2.4%) representing the aminoclay composition whereas phosphorus (4.3%) confirms the presence of DNA in the hybrid. The presence of copper is attributed to the carbon mesh film on copper grid.

Zeta potential of aminoclay (100 mg/mL), DNA (150 ng/µL), and DNA and aminoclay mixture (150 ng/µL in 100 mg/mL of aminoclay solution) showed +35.0±3.5 mV, −24.6 mV±5.2, and +26.2±5.1 mV, resulting in 49.87±5.1 nm, 466.53±20.34 nm, and 550.33±7.8 nm of hydrodynamic diameter size in aqueous solution, respectively. TEM analyses were performed (**Figure 2**) to ascertain the mechanism of transformation as well to confirm the presence of DNA in the nanostructure. TEM analysis confirmed that the organo-building blocks of aminoclay and plasmid DNA molecules, undergoes an electrostatic attraction that favors the formation of hybrids (**Figure 2a and b** **)**. Single molecules of plasmid DNA were condensed by coating with aminoclay, forming nanostructures of 10.5–11.5 nm in diameter and ∼2 µm in length (**Figure 2c** **)**, corresponding to size reduction of hydrodynamic diameter in DNA and aminoclay mixture by DLS method. These observations were confirmed by elemental analysis (**Figure 2d** **)** in which P indicates the presence of DNA molecules and Mg, Si, and Cl indicates elements derived from aminoclay, confirmation by comparison of only aminoclay (Figure S4 in File S1). Aminoclay-sheathed DNA hybrids can be easily adsorbed onto the cell [15], [23], [35], and upon application of physical forces, can penetrate into the intact microalgal cells, resulting in successful integration of donor DNA into the nuclear host DNA.

### Optimal conditions of transformation

**Figure 3:**
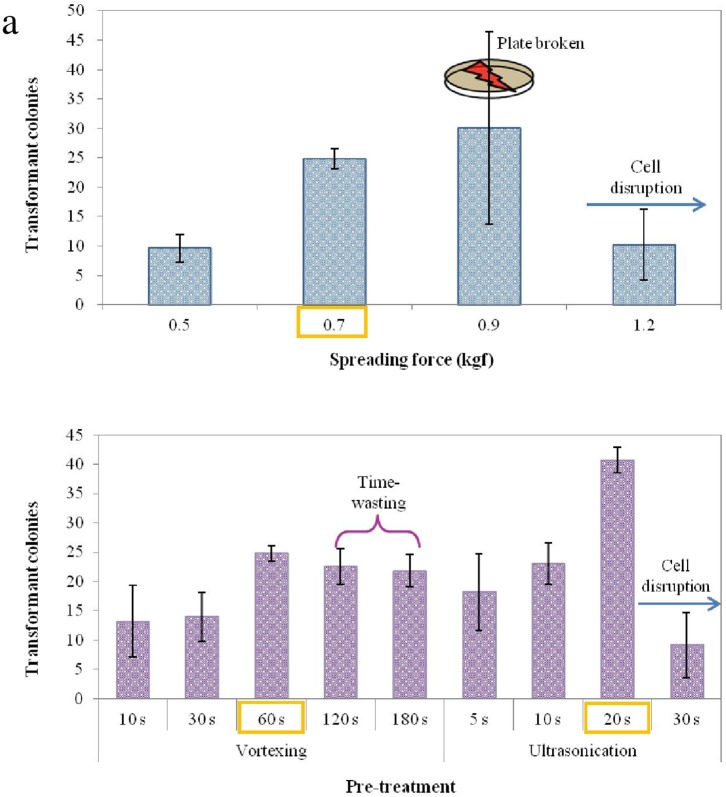
Optimal conditions for transformation. (a) effect of perpendicular force for spreading friction where the plate broken at 9 kgf led to the large error, (b) influence of pre-treatment processes and time. Data are expressed as mean ± standard deviation from three independent experiments. A one-way analysis of variance with Tukey’s multiple comparisons was performed for comparison with the difference between treatment and control groups. Differences were considered statistically significant at *p*<0.05.

As ascertained in earlier experiments, friction force seemed to be an important parameter in facilitating cell rupture, however, further increase in friction force resulted in damage of agar surface and resultant false colonies or smear. In order to apply the maximum friction force to the agar plates, a 4% concentration of agar was chosen based on mechanical strength of the agar medium as well as high transformation efficiency (Figure S5 in File S1) in which as increase of agar loading, it was increased in springiness, hardness (g), and gumminess (g), indicating enhancement of elasticity and stickiness. Notably, at concentrations above 4% of agar resulted in insufficient mixing of antibiotic and nutrients, and impairment in growth in some parts of the plate. Subsequently, spreading time was also standardized at 4 minutes (**Figure 3a and b**). All parameters were further optimized including pre-treatment methods, spreading volume (µl), and agar concentration (%), as summarized in Table S1 in File S1 and the statistical significance of each parameter was computed using multiple regression analysis (SPSS Statistics program, ver. 10).

**Figure 4:**
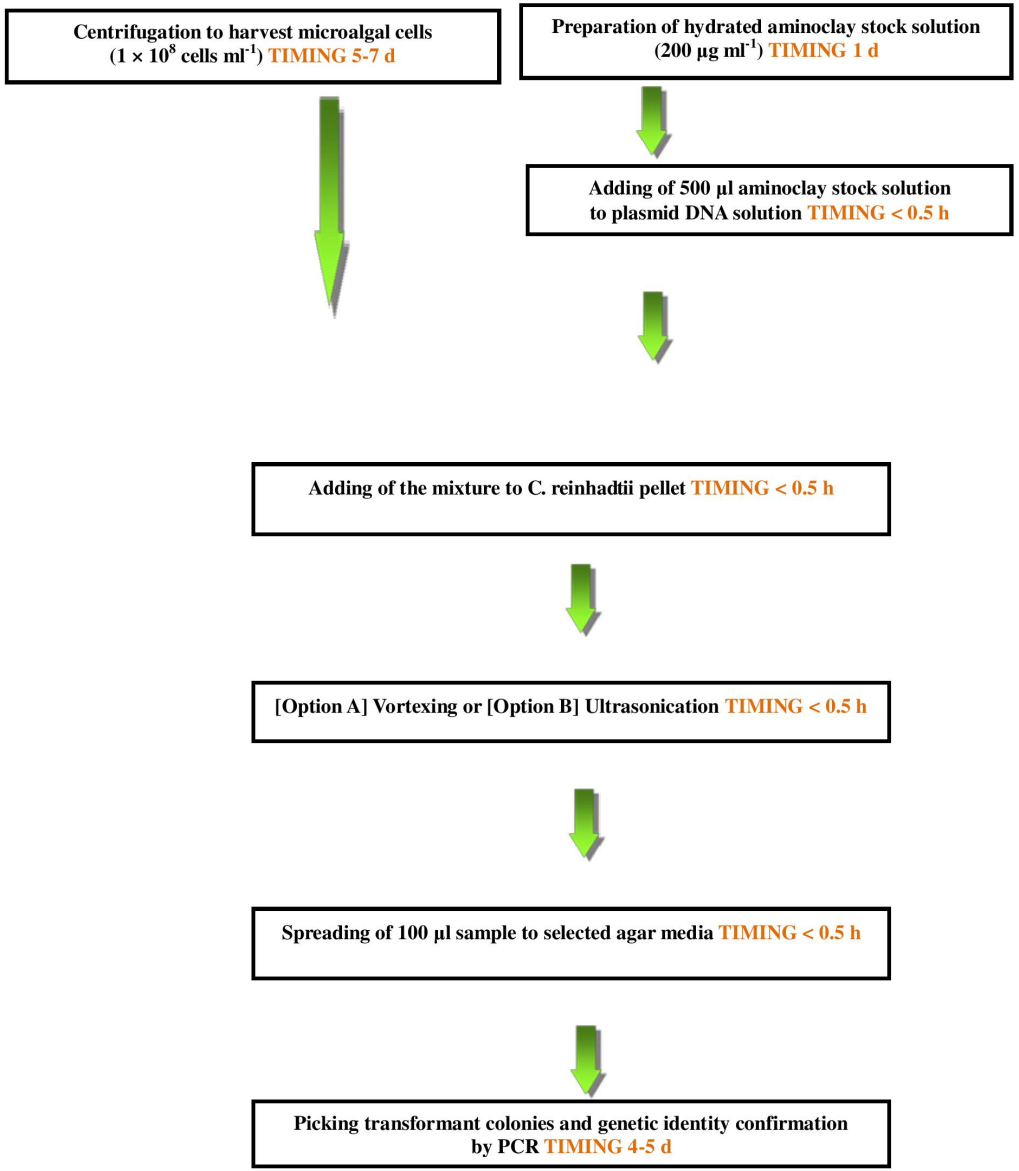
Flow diagram showing an overview of the aminoclay nuclear transformation method.

Nevertheless, an important parameter which yielded the transformants was not quantified and therefore not optimized. Hence, a mechanical spreading device was constructed which would not only quantity spreading force but also yield reproducible results by eliminating manual errors (**Figure 4**). The instrument developed can adjust both spreading acceleration (rpm) and mass employed, however, standardization was achieved by changing the spreading acceleration with mass being kept constant. A pictorial representation of the standardized protocol has been given in **Figure 4**. Using **Option A** we obtained ∼25 colonies (corresponding to frequency of 4.0×10^−6^/cell), by vortexing for 60 seconds, adding 100 µl mixture of plasmid DNA-aminoclay solution and cells under 0.7 kgf force (**Figure 3**). Using **Option B**, with tip ultrasonication for 20 seconds, and adding 300 µl mixture of plasmid DNA-aminoclay solution and cells under 0.9 kgf force, we obtained ∼40 colonies (corresponding to frequency of 2.5×10^−5^/cell) (Figures 3 and Figure S2c in File S1).

### Confirmation of transformation

**Figure 5:**
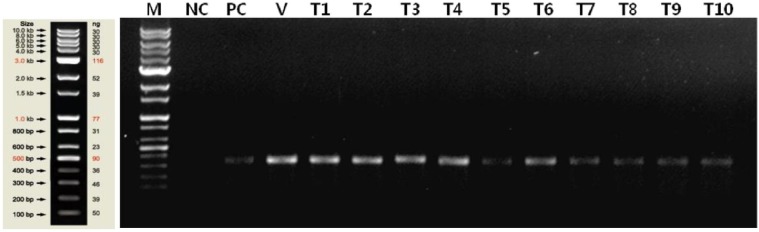
Gel-electrophoresis of PCR products of transformant colonies. PCR products were loaded onto a 2% agarose gel. M: 1 kb ladder (Solgent); NC: negative control (*C. reinhardtii* CC-124); PC: positive control (TR01); V: pCR102 vector; T1–T10: transformants.

Using PCR, we confirmed the genetic identity of ten transformed colonies obtained by optimal vortexing pre-treatment (**Figure 5**). The data show the amplification of a 341 bp product of Hygromycin B gene in the selected transformants. The transformed DNA was randomly integrated into the nucleus without severe damage to the cells [36].

For Soutrhern blot anlaysis (Figure S6 in File S1), Figure S6a in File S1 showed the positions of the probe and the restriction enzyme sites in the pCR102 vector. In Figure S6b in File S1, Southern blotting of transgenic lines (T1 and T2) which were transformed with the pCR102 vector and found to have an aph7” after screening by PCR. Genomic DNA was digested with *Kpn* I and probed with a radiolabeled DNA fragment. Mutant T1 and T2 show a single band, corresponding to a unique integration site of the vector.

## Discussion

Although confirmation of transformation and transformants were obtained using aminoclay, the mechanism of transformation and formation of a hybrid nanostructure involving DNA and aminoclay was not established.

As mentioned before, TEM analysis confirmed that both an effective coating of aminoclay and cell wall rupture is important for efficient transformation of microalgal cells (**Figures 1** and **2**). The hydrodynamic diameter size of aminoclay shows averaged 49.87±5.1 nm with a mono-distribution, which was widely ranging from 20 nm to 70 nm. In addition, TEM measurement of aminoclay sizes (Figure S4 in File S1) exhibits in single- and a few-layered 30 to 150 nm in size by delamination, corresponding to the previous literature [37]. Usually, nanoparticle’s size of uptake in microalgal cells was known to 5–20 nm [38]. Because bigger size of aminoclay cannot easily penetrated into the two cell’s barriers (i.e., cell wall and plasma membrane), the spreading friction damaged cell wall to produce holes on cell walls, resulting in DNA-aminoclay mixture reached into the nuclear region. As a result, larger sized cationic nanoparticles (>100 nm) than that of aminoclay particles might not be expected to play a significant role in invasion of DNA into the internal microalgal cell, depending on cell size and structure. In this respect, the positively charged aminoclay also facilitated the adsorption onto cell walls and then penetration into the internal cells, which rendered to positively charged DNA-aminoclay mixture (+26.2±5.1 mV). DNA was coated or wrapped with organo-building blocks of aminoclay where protonated aminopropyl side chains in aminoclay strongly interacted negatively charged DNA by electrostatic attraction, showing ultrathin armored DNA structure (**Figure 2**).

**In** **Figure 3a**, Taking into the consideration of agar’s elasticity and stickiness, the applied friction fore was ∼7 kgf force. Above this force value, agar dish was slightly broken and some microalgal cells positioned into damaged holes or periphery on petrdish, granting no suitable friction force onto the cells. For the pre-treatments of vortexing and tip ultrasonication for nuclear transformation efficiency (**Figure 3b**), votexing and tip ultrasonication of DNA-aminoclay mixtures showed 30 s and 20 s, respectively. The nuclear transformation efficiency by vortexting at>30 s was moderate results while tip ultrasonication at >20 s resulted in additional damaged cells with losing their healing ability. The nuclear transformation efficiency of tip ultrasonication pretreatment for 20 s exhibited about 2 folds efficiency than that of vortexing pretreatment for 30 s, indicating strong energy based mixing of DNA, aminoclay, microalgal cells is better transformation condition. Notably, TEM analysis also revealed that ∼5–10% of cells was permanently damaged by the treatment and were identified as dead cells, thereby effecting transformation efficiencies.

The aminoclay method is one of the most efficient methods for transformation and is non-sophisticated (**Table 1**). The advantages and limitations of other conventional transformation methods (**Table 2**) as well as the limitations and troubleshooting for aminoclay transformation (Table S2 in File S1) have been summarized. The aminoclay method overcomes several limitations of the conventional methods and advantages of aminocaly method include: (1) ease of preparation of aminoclay, which can be performed at room temperature by one-pot sol-gel reaction and potentially scaled up for mass-production [22], [28], [31], (2) aminoclays are environmentally safe nanomaterials with minimal ecotoxicological effects [30] and no cytotoxicity [25]. (3) cationic-charged aminoclays destabilize intact microalgal cells within a short time-frame by ensheathing the cell surface while preserving cell viability [23], [24], [39], rendering the cell walls permeable to plasmid DNA upon application of physical force. (4) aminoclay-coated DNA molecules are reported to be protected from enzymatic cleavage in the cytoplasm and on the cell surface [13], [15], further improving the access of the donor DNA to the nucleus. (5) aminoclays exhibit antibacterial and antifungal activities under experimental conditions [20], [21], which may lower the possibility of bacterial contamination during the transformation procedure. Finally, (6) a further DNA extraction step can be performed in the presence of 0.2 wt% of aminoclay, which facilitates destabilization of cell walls during the bead-beating process [40].

**Table 1:** Comparison of nuclear transformation efficiency for *C. reinhardtii.*

Method	Total DNA (µg)	Mix volume (µℓ)	Plated DNA (µg)	Colonies	Efficiency (cells/µg DNA)	Repetition	References
Glass bead	2.0 µg	250–300 µℓ	1.0 µg	42	4.20E+01	>3	[42]
Bombardment	0.8 µg	400 µℓ	0.8 µg	2.5	3.13E+00	>3	[43]
Electroporation	2.5 µg	250 µℓ	1.0 µg	1200	1.20E+03	3	[44]
Aminoclay	1.5 µg	500 µℓ	0.3 µg	151	5.03E+02	5	This study

Note: Relative transformation efficiencies of glass bead, bombardment, and electroporation for aminoclay method are calculated to be 0.835, 0.006, and 2.386, respectively.

**Table 2:** Advantages and disadvantages of current nuclear transformation methods for eukaryotic microalgae.

Methods	Advantages	Disadvantages	References
Glass beads	Simple, no need ofexpensive transgenicequipment	Immature protoplastregeneration technique	[3], [4], [7]
Silicon carbon whiskers	No need ofpermeabilization step,cost-effective	Must be performedunder strict safeguardfor inhalation hazard	[5], [6]
Electroporation	Simple, universalusage to different genera	Constrained inbrown algae	[5]–[7], [9]
Biolistic transformation	Possibility of diversifiedvectors usage,controllable andmature manipulation	Need of specializedand high cost equipment	[5]–[7]
*Agrobacterium* *tumefacines*-mediatedgenetic method	No need ofpermeabilization step	Highly dependenton many elements,technically challenging	[5]–[8], [10]

## Conclusion

In précis, the aminoclay-based nuclear transformation protocol for intact microalgal cells that we present here is a very simple, efficient, safe, and reproducible. Microalgal transformation with this fast and effective method could hasten both basic and applied research in microalgal biotechnology. Further studies are being performed to demonstrate the applicability of the method into other important strains such as *Neochloris* and *Nannochloropsis*. or *Chlorella* sp. [41].

## Supporting Information

File S1 **Supporting Information.** Method S1, List of materials and equipments. Text S1, Description of reagent setup. Table S1, Summary of the optimal conditions established for nuclear transformation of *C. reinhardtii.* Table S2, Limitations and its solution of transformation by aminoclay. Figure S1, Schematic representation of spreading friction equipment. Figure S2, Digital camera images of resultant transformant colonies. Figure S3, Schematic representation of the approximate unit structure of aminoclay ([H_2_N(CH_2_)_3_]_8_Si_8_Mg_6_O_12_(OH)_4_)). Figure S4, TEM images and energy dispersive X-ray (EDX) analysis of aminoclay. Figure S5, Mechanical properties of plates with increasing concentration of agar (1.5%, 3.0%, and 4.0%). Figure S6, Schematic diagram of an expression construct and Southern blot analysis.
(DOC)
