# Supplementary material for: A Simple and Non-Invasive Method for Nuclear Transformation of Intact-walled Chlamydomonas reinhardtii
Source: PLoS One. 2014 Jul 2;9(7):e101018. doi: 10.1371/journal.pone.0101018 (PMC4079685; doi:10.1371/journal.pone.0101018)
Supplement: File S1 — Supporting Information. Method S1, List of materials and equipments. Text S1, Description of reagent setup. Table S1, Summary of the optimal conditions established for nuclear transformation of C. reinhardtii. Table S2, Limitations and its solution of transformation by aminoclay. Figure S1, Schematic representation of spreading friction equipment. Figure S2, Digital camera images of resultant transformant colonies. Figure S3, Schematic representation of the approximate unit structure of aminoclay ([H2N(CH2)3]8Si8Mg6O12(OH)4)). Figure S4, TEM images and energy dispersive X-ray (EDX) analysis of aminoclay. Figure S5, Mechanical properties of plates with increasing concentration of agar (1.5%, 3.0%, and 4.0%). Figure S6, Schematic diagram of an expression construct and Southern blot analysis. (DOC) [file pone.0101018.s001.doc]

**Supporting Information**

**A Simple and Non-invasive Method for Nuclear Transformation**

**of Intact-walled *Chlamydomonas reinhardtii***

**Method S1**

**List of materials and equipments**

*1. For synthesis of aminoclay*

• Magnesium chloride hexahydrate (MgCl_2_•6H_2_O; Sigma-Aldrich, cat. no. 7791-18-6)

• (3-Aminopropyl)triethoxysilane (C_9_H_23_NO_3_Si; Sigma-Aldrich, cat.no.919-30-2) • Ethanol (C_2_H_5_OH; 95.0%, Samchun Pure Chemicals, cat.no. 64-17-5, 18L)

*2. For C. reinhardtii culture*

• Ammonium chloride (NH_4_Cl; JUNSEI, CAS no. 12125-02-9)

• Magnesium sulfate heptahydrate (MgSO_4_•7H_2_O; JUNSEI, CAS no. 10034-99-8)

• Calcium chloride dihydrate (CaCl_2_•2H_2_O; JUNSEI, CAS no. 10035-04-8)

• Dipotassium phosphate (K_2_HPO_4_; Samchun Pure Chemicals, CAS no. 7758-11-4)

• Monopotassium phosphate (KH_2_PO_4_; Samchun Pure Chemicals, CAS no. 7778-77-0)

• EDTA disodium salt dihydrate (C_10_H_14_N_2_Na_2_O_8_•2H_2_O; Sigma-Aldrich, CAS no. 6381-92-6)

• Zinc sulfate heptahydrate (ZnSO_4_•7H_2_O; JUNSEI, CAS no. 7446-20-0)

• Boric acid (H_3_BO_3_; JUNSEI, CAS no. 10043-35-3)

• Manganese chloride tetrahydrate (MnCl_2_•4H_2_O; Sigma-Aldrich, CAS no. 13446-34-9)

• Cobalt chloride hexahydrate (CoCl_2_•6H_2_O; Sigma-Aldrich, CAS no. 7791-13-1)

• Copper sulfate pentahydrate (CuSO_4_•5H_2_O; Sigma-Aldrich, CAS no. 7758-99-8)

• Ammonium molybdatetetrahydrate ((NH_4_)_6_Mo_7_O_24_•4H_2_O; Sigma-Aldrich, CAS no. 12054-85-2)

• Ferrous sulfate heptahydrate (FeSO_4_•7H_2_O; Sigma-Aldrich, CAS no. 7782-63-0)

• Tris ((HOCH_2_)_3_CNH_2_; Bio-Rad Laboratories, CAS no. 77-86-1)

• Glacial acetic acid (CH_3_CO_2_H; Sigma-Aldrich, CAS no. 64-19-7)

• Hygromycin B (C_2_OH_37_N_3_O_13_; Duchefa Biochemie B.V., CAS no. 31282-04-10)

• Whatman® grade #1 filter paper (55 Φ mm, CAS no. 1001-055)

• White light (DULUX L 36W/954, OSRAM, Korea)

*3. For C. reinhardtii transformation*

• Synthesized aminoclay

• Hygromycin B (C_2_OH_37_N_3_O_13_; Duchefa Biochemie B.V., CAS no. 31282-04-10)

• pCR102 (6,046 bp) after plasmid DNA extraction

• Vector (1.3 kb) carrying the hygromycin resistance aph7’’ gene

• 1.5 ml-Eppendorf tubes (Greiner Bio-One, CAS no. 616-201, Frickenhausen, Germany)

• 60 mm diameter Petri dishes (Greiner Bio-One, Frickenhausen, Germany)

• Sepiolite (Mg_2_H_2_(SiO_3_)_3_•xH_2_O, Sigma-Aldrich, CAS no. 63800-37-3)

*4. For DNA extraction*

• 0.5 mm Zirconia/Silica bead (BioSpec Products, Inc., USA)

• i-genomic DNA Extraction Mini Kit for Plant (iNtRON Biotechnology, Korea)

*5. For PCR analysis*

• Agarose (Bio-Rad Laboratories, CAS no. 9012-36-6)

• TBE (Tris-borate-EDTA) buffer (iNtRON Biotechnology,)

• Dimethyl sulfoxide (DMSO, (CH_3_)_2_SO; Sigma-Aldrich, CAS no. 67-68-5)

• Betaine ((CH_3_)_3_N^+^CH_2_COO^-^; Sigma-Aldrich, CAS no.107-43-7)

• Primers: hpt204-L (Forward primer (5'-AGCTACCGCTTCAGCACTTG-3')) and hpt1579-R (Reverse primer (5'-TGGTGCAACTGCATCTCAAC-3')) (Cosmo genetech; individual order)

• Plasmid DNA Purification Kit (iNtRON Biotechnology, Korea)

• Wizard SV Gel and PCR Clean-Up System (Promega, USA)

*6. For Transmission Electron Microscopy (TEM) examination*

• Carbon 300 mesh film on square copper grids (Electron Microscopy Sciences, CAS no.71150)

• Paraformaldehyde (HO(CH_2_O)_n_H; Electron Microscopy Sciences, CAS no.19210)

• Glutaraldehyde solution (25% OHC(CH_2_)_3_CHO for grade in H_2_O; Sigma-Aldrich, CAS no.111-30-8)

• Osmium tetroxide solution (4% OsO_4_ in H_2_O for electron microscopy; Sigma-Aldrich, CAS no. 20816-12-0)

• Uranyl acetate dihydrate (UO_2_ (CO_2_H_3_)_2_•2H_2_O; Fluka, CAS no.6159-44-0)

• Lead (Ⅱ) citrate tribasictrihydrate ((C_6_H_5_O_7_)_2_Pb_3_•3H_2_O for electron microscopy; Sigma-Aldrich, CAS no.107-43-7)

• Ethanol (C_2_H_5_OH, absolute for analysis, MerkKGaA, Index no. 603-002-00-5, Germany)

• Propylene oxide solution (C_3_H_6_O; Sigma-Aldrich, CAS no.75-56-9)

• Epon-812 resin (Electron Microscopy Sciences, CAS no.1232)

*7. Spreading friction apparatus*

• Glass spreader (4 mm diameter glass rod with polished ends with 130 mm long handles and 50 mm long spreader segment)

• Turntable for Petri dishes (schuettpetriturn-E, electrically driven, 230 V, CAS no. 3.361 252)

• Experimental marble stand (DH.KA11176, W300 × D200 × H600 mm, SUS Rod Φ9, DAIHAN )

• Force gauge (FD102, 0.25 kgf-2.5 kgf, Mecmesin)

• A level (Z-340, HOZAN)

*8. Commercially available kits*

• Plasmid DNA Purification Kit (iNtRON Biotechnology, Korea)

• i-genomic DNA Extraction Mini Kit for Plant (iNtRON Biotechnology, Korea)

•Wizard SV Gel and PCR Clean-Up System (Promega, USA)

**TEXT S1**

**Reagent setup**

Tris-Acetate-Phosphate (TAP) salts solution [1] was prepared by dissolving 15.0 g NH_4_Cl, 4.0 g MgSO_4_•7H_2_O, 2.0 g CaCl_2_•2H_2_O in double-distilled water (DI water) and, then, making up to 1,000 ml. For 1× PBS solution, it was prepared by dissolving 28.8 g K_2_HPO_4_, 14.4 g KH_2_PO_4_ in DI water and making up to 1,000 ml. The following reagents were dissolved in 50 ml DI water for Hutner’s trace elements. Briefly, prior to 1 liter final mixture solution, prepare 50.0 g C_10_H_14_N_2_Na_2_O_8_•2H_2_O, 22.0 g ZnSO_4_•7H_2_O, 11.4 g H_3_BO_3_, 5.06 g MnCl_2_•4H_2_O, 1.61 g CoCl_2_•6H_2_O, 1.57 g CuSO_4_•5H_2_O, 1.10g (NH_4_)_6_Mo_7_O_24_•4H_2_O, 4.99 g FeSO_4_•7H_2_O. Mix together all solutions with exception of EDTA. Boil and then add EDTA solution. The mixture should turn green. When they are fully dissolved, cool the mixture to 70°C. Keep temperature at 70°C, and add 85 ml of hot 20% KOH solution (0.5g ml^-1^ final volume). Bring the final solution to 1 liter total volume, by adding DI water. This solution should be clear green initially. Cap the flask with a cotton plug and let it stand for 1-2 weeks, shaking it once a day. The solution should eventually turn purple and leave a rust-brown precipitate, which can be removed by filtering through two layers of Whatman #1 filter paper. If no precipitate forms, the solution is usable anyway.

For liquid TAP medium, mix 25.0 ml of TAP salts solution with 0.375 ml PBS buffer, 1.0 ml Hutner’s trace elements, 1.0 ml glacial acetic acid, 2.42 g (HOCH_2_)_3_CNH_2_. Adjust to pH 7.0 with KOH or HCl and make up to 1,000 ml. The reagents should be mixed in a bottle big enough to not be filled for more than two-thirds. Cover with aluminum foil and autoclave at 121 °C for 15 min. For solid TAP medium, mix 15.0 g Bacto agar, with 25.0 ml TAP salts solution, 0.375 ml PBS buffer 1.0 ml Hutner’s trace elements, 1.0 ml glacial acetic acid, and 2.42 g Tris. Adjust pH to 7.0 with KOH or HCl and make up to 1,000 ml. The reagents should be mixed in a flask large enough so that liquid does not exceed more than two-thirds of the volume. Cover with aluminum foil and autoclave at 121 °C for 15 min. After cooling down to 60 °C, pour 25 ml medium per plate whose plates are sterile plastic Petri dishes under aseptic conditions.

For medium A (4% agar with Hygromycin B) for selection of transformants, briefly, mix 45.0 g Bacto agar with 0.02 g Hygromycin B, 25.0 ml TAP salts solution, 0.375 ml PBS buffer solution, 1.0 ml Hutner’s trace elements, 1.0 ml glacial acetic acid, and 2.42 g Tris. Adjust pH to 7.0 with KOH or HCl and, then, make up to 1,000 ml. The reagents should be mixed in a flask large enough so liquid volume does not exceed two-thirds. Cover with aluminum foil and autoclave at 121 °C for 15 min. After cooling down to 60 °C, add hygromycin B and pour the medium, 25 ml medium per plate, in sterile plastic Petri dishes under aseptic conditions. Let the agar plates to dry at room temperature and cover with aluminum foil. Hygromycin B is sensitive to light and heat, thus the medium must be cooled to approximately 60 °C before adding the antibiotic and the supplemented medium, and stored at 4 °C in the dark. To prepare medium B (1.5% agar with Hygromycin B) for purification and short-term storage of transformants, mix 15.0 g Bacto agar with 0.02 g hygromycin B, 25.0 ml TAP salts solution, 0.375 ml PBS buffer, 1.0 ml Hutner’s trace elements, 1.0 ml glacial acetic acid, and 2.42 g Tris. Adjust pH to 7.0 with KOH or HCl and, then, make up to 1,000 ml with DI water. The reagents should be mixed in a flask large enough so liquid volume does not exceed two-thirds. Cover with aluminum foil and autoclave at 121 °C for 15 min. After cooling down to 60 °C, add hygromycin B and pour 25 ml medium per plate, in sterile Petri dishes under aseptic conditions. Let the agar plates dry at room temperature and cover with aluminum foil.

**Reference**

1. Gorman DS, Levine RP (1965) Cytochrome f and plastocyanin: their sequence in the photosynthetic electron transport chain of *Chlamydomonas reinhardii*. Proc Natl Acad Sci USA 54: 1665-1669.

**Table S1**

Summary of the op*timal conditions establi*shed *for nuclear transformation of C. reinhardtii*.

| Pre-treatment | Vortexing for 60 s  (**Option A**) | Tip ultrasonication for 20 s  (**Option B**) |
| --- | --- | --- |
| Spreading force (kgf) | 0.7 | 0.9 |
| Spreading volume (μl) | 100 | 300 |
| Spreading time (min) | 4 | 4 |
| Agar concentration (%) | 4 | 4 |

Table S2

Limitations and its solution of transformation by aminoclay.

| **Problem** | **Possible reason** | **Solution** |
| --- | --- | --- |
| Bacterial and fungal contaminations | No sterile conditions, careless handling | Use aseptic culture conditions  Cultivate cells with 2 g/L of aminoclay (Farooq *et al.*, 2013) |
| Rupture of agar plate surfaces during the spreading step | Low percentage  of agar content,  high spreading force | Adjust to maximum 4% agar plate.  Load < 0.9 kgf spreading force. |
| No transformants colonies | Significant damage or disruption of the cells, excess concentrations of antibiotics | Load < 0.9 kgf spreading force.  Treat the cells with appropriate concentrations of antibiotics. |
| Unfavorable DNA extraction | Thick cell walls | Increase the time of bead-beating. Inject a 0.2 wt% of hydrated aminoclay while bead-beating (Kim *et al.*, 2013) |


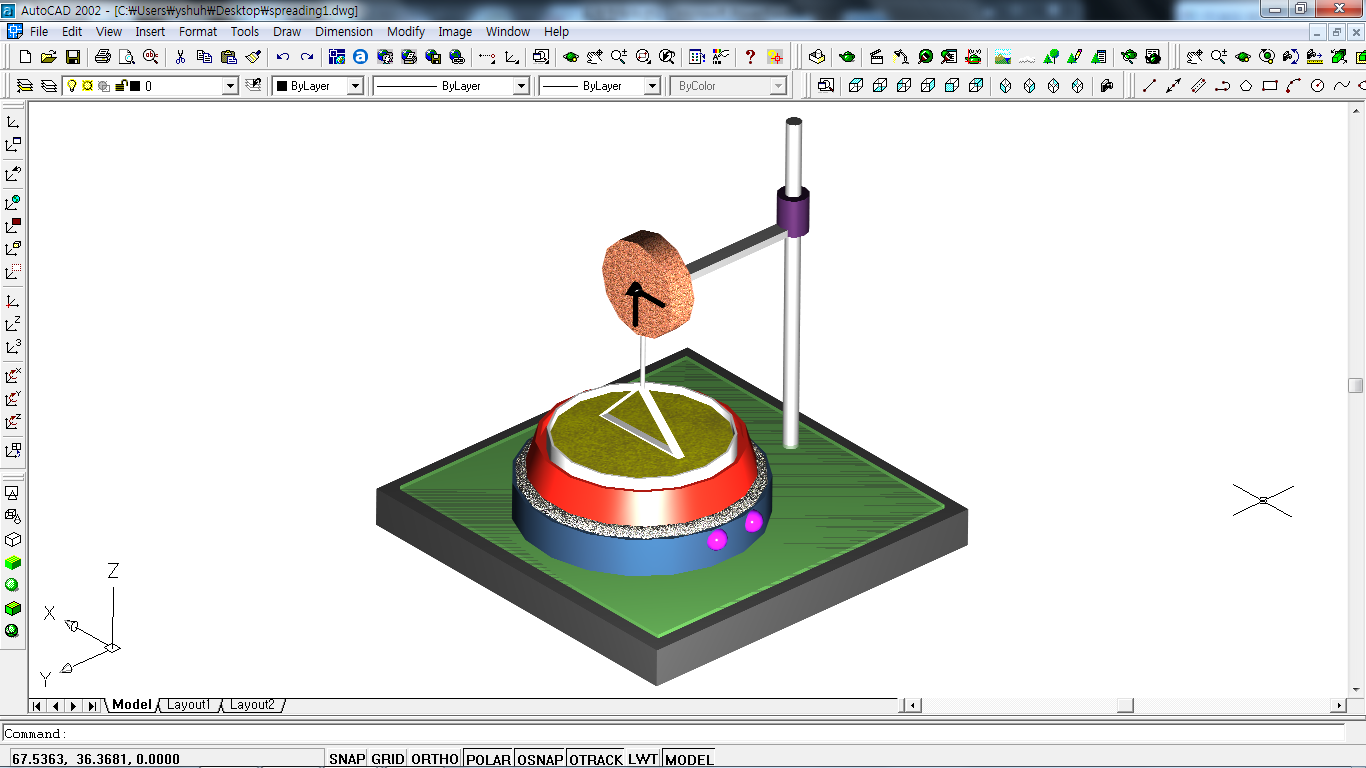


[1]

[7]

[2]

[3]

[4]

[5]

[6]

Figure S1. Schematic representation of spreading friction equipment used in our laboratory. [1]: Perpendicular force gauge, [2]: Triangular-shaped stir stick, [3]: Agar plate, [4]: Turntable, [5]: Speed button, [6]: Timer button, [7]: Experimental marble stand.


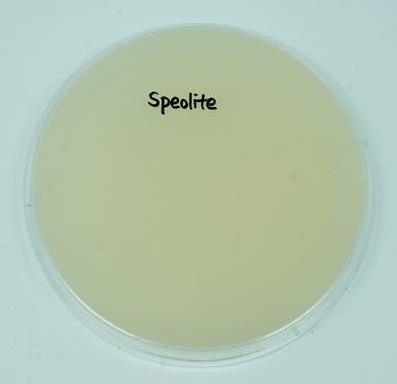


**Sepiolite**

a


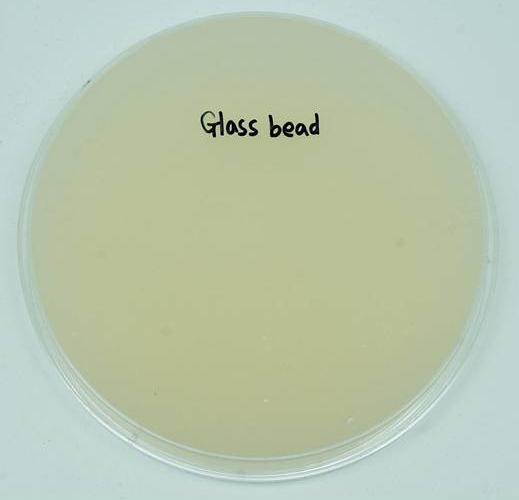


**Glass bead**

b

c


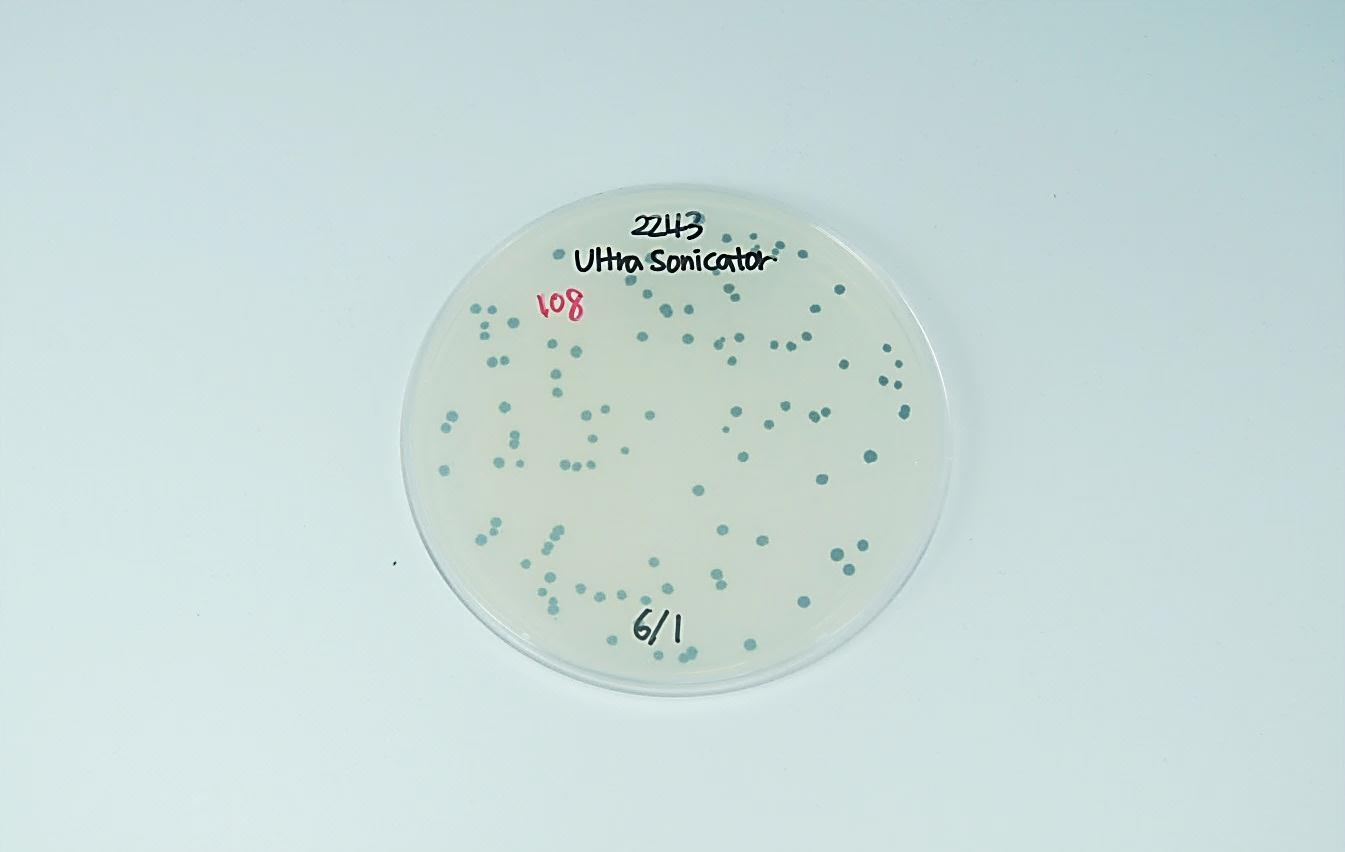


**Ultrasonication**

Figure S2. Digital camera images of resultant transformant colonies. (a) Sepiolite (Yosida effect) method, (b) glass bead stirring, and (c) Option B method grown on selective medium A for 7 days at 25 °C.


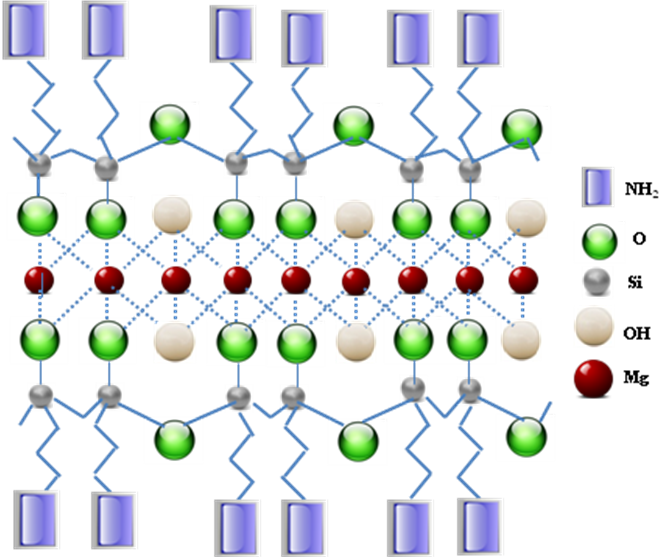


**Figure S3. Schematic representation of the approximate unit structure of aminoclay ([H_2_N(CH_2_)_3_]_8_Si_8_Mg_6_O_12_(OH)_4_))**. Reproduced with permission of references [21-23].


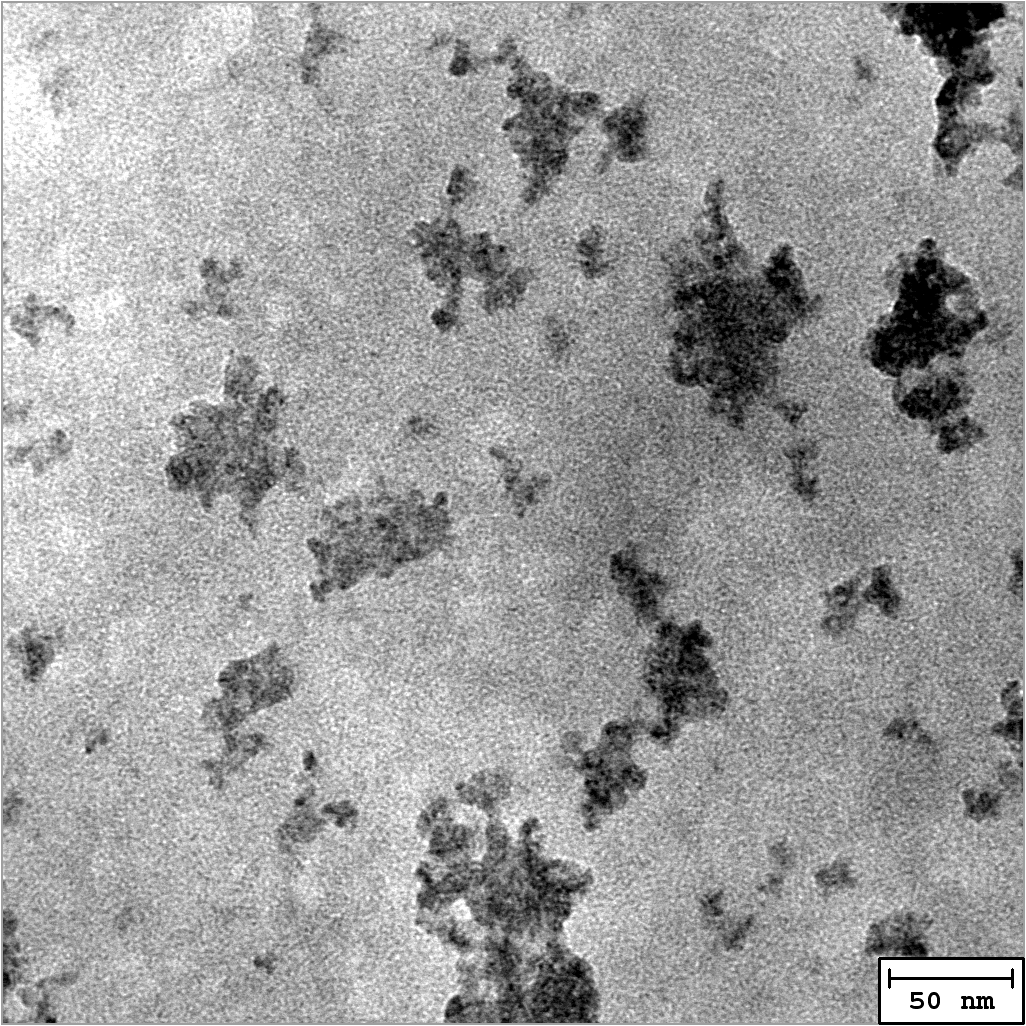

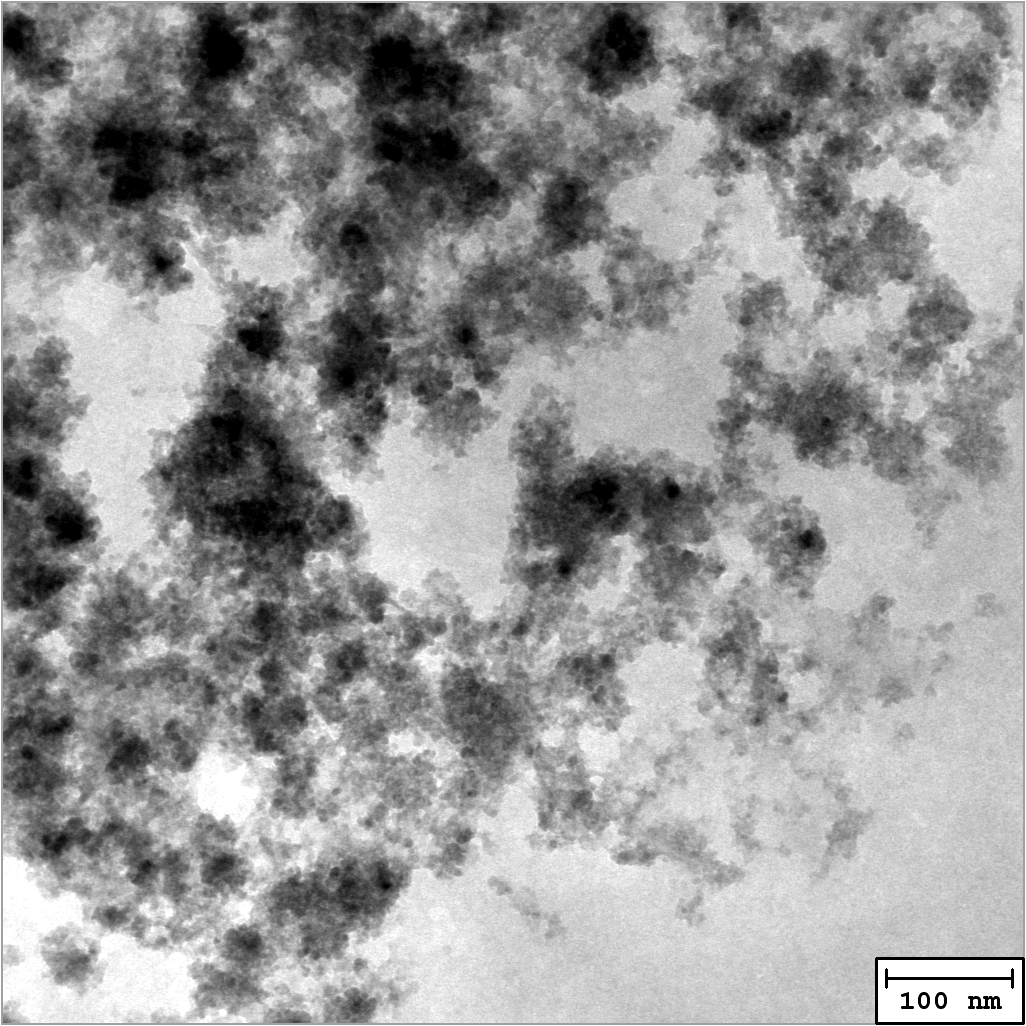


a

b

**
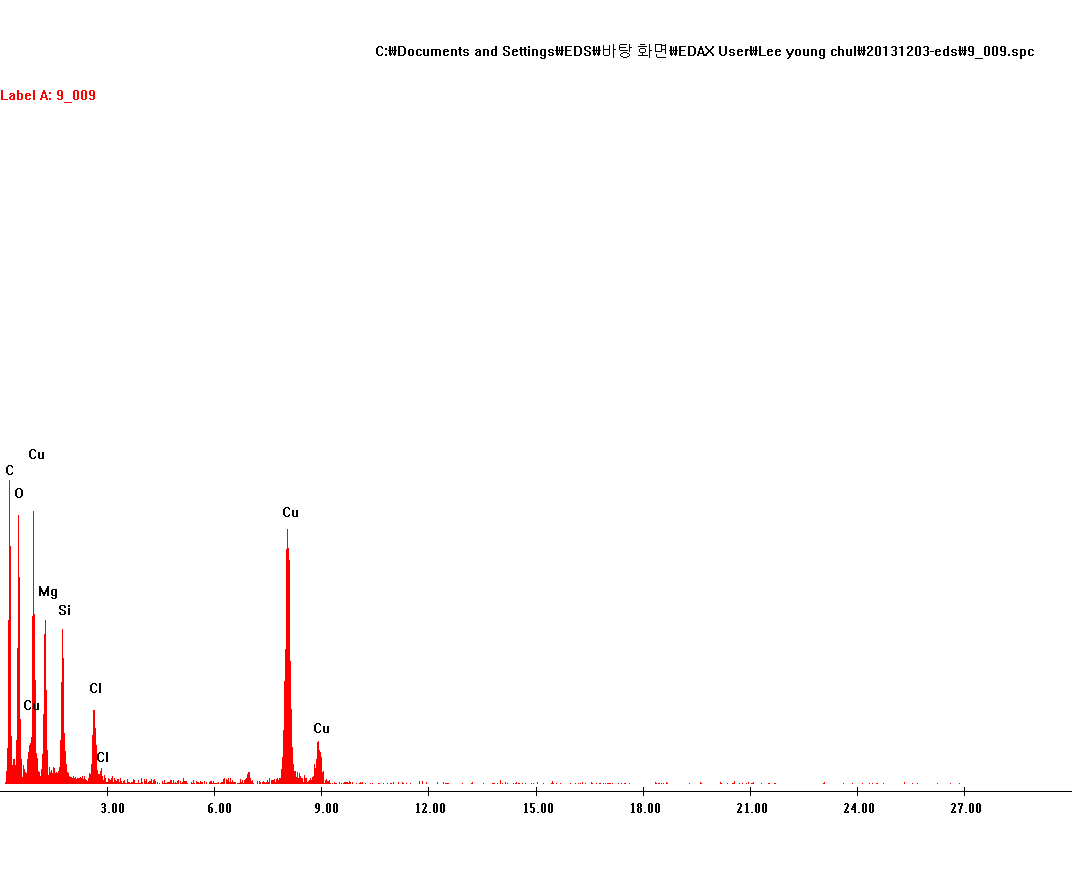
**

c

**Figure S4. TEM images and composition analysis**. (a,b) Electron micrographs of aminoclay (0.25 mg/mL) in DI water and (c) Energy dispersive X-ray (EDX) analysis.

a

b

c

**Figure S5. Mechanical properties of plates with increasing concentration of agar (1.5%, 3.0%, and 4.0%)**. (a) Springiness, (b) Hardness, and (c) Gumminess. Data are expressed as mean ± standard deviation from three independent experiments.


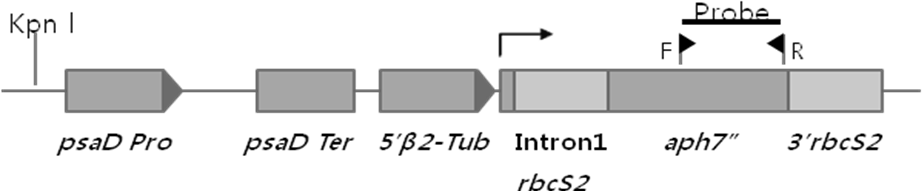


a


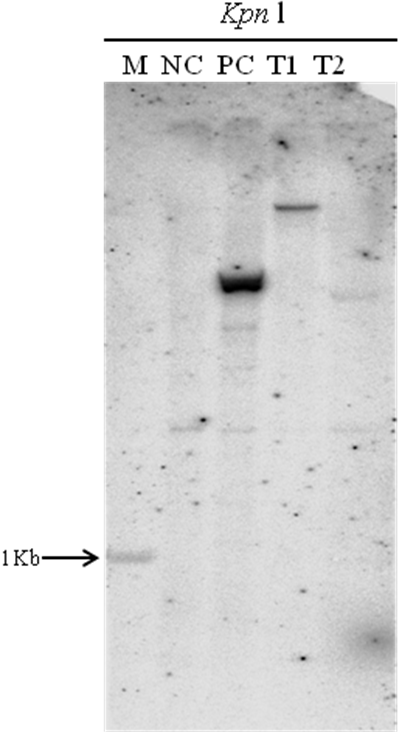


b

Figure S6. Southern blot analysis of transgenic lines expressing aph7”. (a) Scheme of an expression construct. aph7” primers are indicated by F and R with arrows. (b) Southern blotting of genomic DNA from transgenic lines digested with Kpn I and hybridized with aph7” PCR probe. M: 1kb ladder (Solgent); NC: negative control (*C.reinhardtii* CC-124); PC: positive control (TR01); T1 and T2: transformants.
